# Performance of PRISM III, PELOD-2, and P-MODS Scores in Two Pediatric Intensive Care Units in China

**DOI:** 10.3389/fped.2021.626165

**Published:** 2021-04-28

**Authors:** Lidan Zhang, Yuhui Wu, Huimin Huang, Chunyi Liu, Yucai Cheng, Lingling Xu, Wen Tang, Xuequn Luo

**Affiliations:** ^1^The Pediatric Intensive Care Unit, The First Affiliated Hospital, Sun Yat-Sen University, Guangzhou, China; ^2^Division of Hematology/Oncology, Department of Pediatrics, The Seventh Affiliated Hospital, Sun Yat-Sen University, Shenzhen, China; ^3^The Pediatric Intensive Care Unit, Shen-Zhen Children's Hospital, Shenzhen, China; ^4^The Pediatric Intensive Care Unit, Shenzhen Baoan Maternity and Child Health Hospital, Shenzhen, China; ^5^Department of Pediatrics Hematology, The First Affiliated Hospital, Sun Yat-Sen University, Guangzhou, China

**Keywords:** pediatric intensive care unit, prognostic score, prediction, mortality, cohort study

## Abstract

**Objective:** The performances of the pediatric risk of mortality score III (PRISM III), pediatric logistic organ dysfunction score-2 (PELOD-2), and pediatric multiple organ dysfunction score (P-MODS) in Chinese patients are unclear. This study aimed to assess the performances of these scores in predicting mortality in critically ill pediatric patients.

**Methods:** This retrospective observational study was conducted at two tertiary-care PICUs of teaching hospitals in China. A total of 1,253 critically ill pediatric patients admitted to the two Pediatric Intensive Care Units (PICUs) of the First Affiliated Hospital, Sun Yat-Sen University from August 2014 to December 2019 and Shen-Zhen Children's Hospital from January 2019 to December 2019 were analyzed. The indexes of discrimination and calibration were applied to evaluate score performance for the three models (PRISM III, PELOD-2, and P-MODS scores). The receiver operating characteristic (ROC) curve was plotted, and the efficiency of PRISM III, PELOD-2, and P-MODS in predicting death were evaluated by the area under ROC curve (AUC). Hosmer–Lemeshow goodness-of-fit test was used to evaluate the degree of fitting between the mortality predictions of each scoring system and the actual mortality.

**Results:** A total of 1,253 pediatric patients were eventually enrolled in this study (median age, 38 months; overall mortality rate, 8.9%; median length of PICU stay, 8 days). Compared to the survival group, the non-survival group showed significantly higher PRISM III, PELOD-2, and P-MODS scores [PRISM III: 18 (12, 23) vs. 11 (0, 16); PELOD-2, 8 (4, 10) vs. 4 (0, 6); and P-MODS: 5 (4, 9) vs. 3 (0, 4), all *P* < 0.001]. ROC curve analysis showed that the AUCs of PRISM III, PELOD-2, and P-MODS for predicting the death of critically ill children were 0.858, 0.721, and 0.596, respectively. Furthermore, in the Hosmer–Lemeshow goodness-of-fit test, PRISM III and PELOD-2 showed the better calibration between predicted mortality and observed mortality (PRISM III: χ^2^ = 5.667, *P* = 0.368; PELOD-2: χ^2^ = 9.582, *P* = 0.276; P-MODS: χ^2^ = 12.449, *P* = 0.015).

**Conclusions:** PRISM III and PELOD-2 can discriminate well between survivors and non-survivors. PRISM III and PELOD-2 showed the better calibration between predicted and observed mortality, while P-MODS showed poor calibration.

## Introduction

The crucial aim of admission to a pediatric intensive care unit (PICU) is to reduce mortality. In this regard, mortality prediction models are important and valuable tools for assessing the quality of care provided to critically ill patients (1). The use of risk-adjustment tools to predict PICU death is a rational and objective approach to quantify severity that has been practiced over the past 20 years (2). The prognostic scores based on these tools can be used for dynamic evaluation of individual pediatric patients and for accurately identifying the risk of death or possible severe complications in critically ill patients of all age groups, including pediatric patients. Based on their predictive functions, these scores can allow timely detection of potential risks, which can then be prevented promptly. At present, the PRISM III, PELOD-2, and P-MODS scores are frequently used for mortality prediction in the pediatric population (3–6). Numerous studies have proven that these scores are valuable risk-adjustment tools, and are therefore applicable for benchmarking the performance of more than 100 PICUs participating in the Virtual PICU System (7). Among them, PRISM III is the most commonly used assessment in Chinese PICUs. PRISM III showed adequate capacity for discriminating between non-survivors and survivors (8). On the other hand, PELOD-2 is the updated version of PELOD and has recently been validated to show sensitive discrimination and good calibration (9). However, at present, there are very limited public data assessing the performance of PELOD-2 in Chinese PICUs.

Considering the dramatic changes in the quality of intensive care as a result of the recent major advances in technology and treatment, the mortality prediction models, including PRISM III, PELOD-2, and P-MODS scores, require reassessment. For optimal use of these scores, their relevance and effectiveness in specific patient populations different from the populations on which they were developed is important. Therefore, this study aimed to explore whether the model (PRISM III, PELOD-2, and P-MODS) could predict the risk of death in two PICUs in China. To this end, we enrolled 1,253 critically ill pediatric patients who were admitted to the PICUs of the First Affiliated Hospital, Sun Yat-Sen University from August 2014 to December 2019 and Shen-Zhen Children's Hospital from January 2019 to December 2019 were analyzed. The mortality and survival rates of the enrolled patients as well as the potential relationship between death and survival were investigated, and the suitability of each score for monitoring the quality of intensive care in our unit was validated.

## Materials and Methods

### Design and Setting

This retrospective observational study was conducted in the pediatric intensive care units of the First Affiliated Hospital, Sun Yat-Sen University from August 2014 to December 2019 and Shen-Zhen Children's Hospital from January 2019 to December 2019.

### Patients

A total of 1,368 consecutive and critically ill pediatric patients were enrolled. There was no children younger than 1 month or older than 14 years in these two PICUs. Patients above 14 years old were admitted to adult ICUs. The following criteria were used to further exclude patients from this study: (1) patients staying in the ICU for <24 h; (2) patients with missing information for the variables used to estimate the PRISM III, P-MODS, and PELOD-2 scores (missing information means that the indicator we had measured, but we were unable to obtain information due to the lack of paper data).

### Methods

The following data were collected for each enrolled patient within 24 h of admission: age, gender, admission diagnosis and all necessary physiological parameters required for estimation of PRISM III, P-MODS, and PELOD-2 scores. For variables that were measured more than once within 24 h of admission, the worst values for the day were selected for calculating the three scores. Moreover, length of PICU stays and clinical outcomes (survival or death) at discharge were also collected.

For the PRISM III score, data for the following 16 variables were collected at 24 h of PICU admission: temperature, systolic blood pressure, heart rate, partial pressure of arterial oxygen (PaO_2_), partial pressure of arterial carbon dioxide (PaCO_2_), Glasgow Coma Scale (GCS) score, pupillary reaction, prothrombin time (PT) and activated partial thromboplastin time (APTT), serum creatinine, serum urea nitrogen, serum potassium, blood glucose, and serum bicarbonate levels, white blood cell and platelet counts.

The P-MODS score can be used to evaluate five body functions, namely, circulation, breathing, liver function, blood coagulation, and kidney function. Because the P-MODS score does not include an assessment of the nervous system, its prognostic value in children with conditions related to the nervous system diseases may be limited.

For the PELOD-2 score, which evaluates five organ systems (neurologic, cardiovascular, respiratory, renal, and hematologic), 10 variables (GCS score, pupillary reaction, lactatemia, mean arterial blood pressures, PaO_2_/FiO_2_ ratio, PaCO_2_, invasive ventilation, creatinine level, white blood cell count, and platelet count) were recorded. It is noted that GCS score was measured only for patients with known or suspected acute central nervous system disease. If the patient was sedated, we recorded the estimated Glasgow Coma Score before sedation. All patients sedated or under surgical anesthesia were considered to have normal GCS score.

To ensure data quality, a contact in charge of data recording and another specified person responsible for supervision were designated in the participating PICUs. To standardize PRISM III, PELOD-2 and P-MODS calculation, an operating procedure manual was created and applied in the participating PICUs. The person in charge was asked to demonstrate the accurate calculation process to the registrar and a senior experienced person. The data were first registered by the registrar and then checked by the senior experienced person. The results were sent to the person responsible for supervision by e-mail. The superintendent collated the data and identified problems in it. The identified problems were discussed and resolved promptly.

### Ethical Considerations

The ethical and scientific aspects of the research were evaluated and approved by the Research Ethics Committees of the participating PICUs. Written informed consent to participate in this study was provided by the participants' legal guardian/next of kin. The data used in the study were protected by patient privacy protection requirements.

### Statistical Analysis

All statistical analyses were performed using Statistical Program for Social Science version 22.0. Categorical variables were expressed as frequencies and percentages. Normally distributed data were expressed as mean [standard deviation (SD)]. The *t* test was applied for comparisons between groups. Non-normally distributed data were expressed as median (interquartile range). A non-parametric rank-sum test was used for comparisons between groups. Counting data were compared by the χ*2* test. To evaluate the discrimination or the ability of the model to differentiate between survivors and non-survivors, the area under the receiver operating characteristic curve (AUC-ROC) and its 95% confidence interval (CI) was calculated. The threshold, sensitivity, and specificity were calculated as well. The threshold with the maximum Youden index was chosen as the best threshold. Acceptable discrimination is represented by an area under the curve of 0.70–0.79, good discrimination by an area ≥0.80, and excellent discrimination by an area ≥0.90 (10, 11). The Hosmer–Lemeshow goodness-of-fit test was used to evaluate calibration or the degree of agreement between the predicted and observed mortality assessed using the PRISM III, P-MODS and PELOD-2 scoring systems. On the basis of the prediction probability, the sample data were divided into 10 groups. *P* values were calculated according to the chi-square distribution of degrees of freedom and the logistic model was validated. If *P* > 0.05, the predicted value of the model was essentially consistent with the observed value, indicating that the predicted model had good calibration; otherwise, if *P* < 0.05, the predicted value of the model was significantly different from the observed value, suggesting that the prognostic model was not effective and had no justification to be applied in that population (12, 13).

## Results

**Figure 1:**
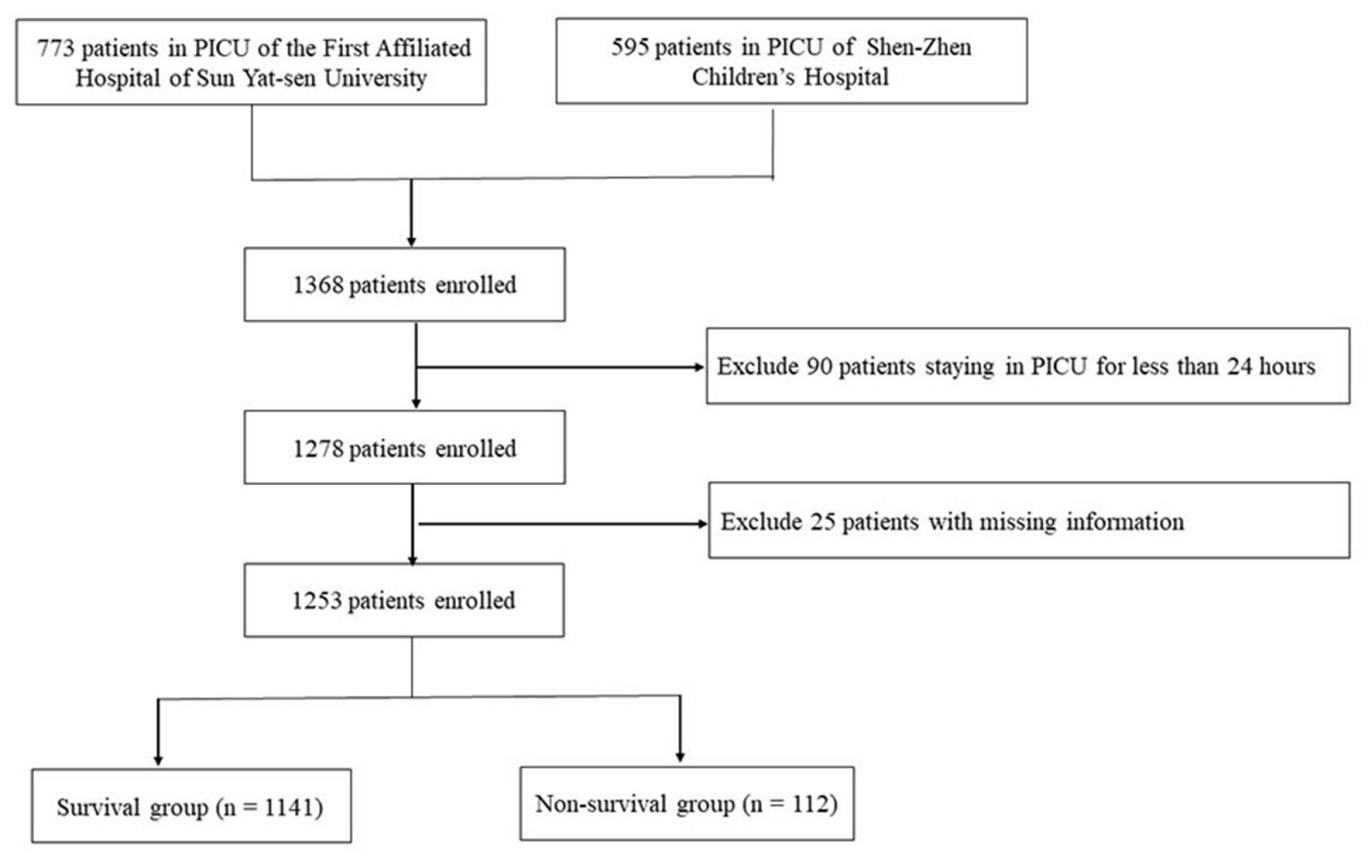
Flow chart of the study population.

A total of 1,368 critically ill pediatric patients admitted in the PICUs of the First Affiliated Hospital, Sun Yat-Sen University (*n* = 773) and Shen-Zhen Children's Hospital (*n* = 595) were initially enrolled. A total of 115 patients were excluded due to the previously described exclusion criteria, including 25 patients with missing data and 90 patients who were hospitalized for <24 h. Thus, a total of 1,253 patients were finally included in this study (Figure 1). Among them, 813 (64.9%) were male and 440 (35.1%) were female; median age was 3.16 (0.68, 8.79) years. Disease categories included infectious disease (169 cases, 13.5%), respiratory disease (327 cases, 26.1%), cancer (205 cases, 16.4%), neurological disease (104 cases, 8.3%), digestive disease (122 cases, 9.7%), urological disease (110 cases, 8.8%), trauma (128 cases, 10.2%), and miscellaneous diseases (88 cases, 7.0%). It is noted that if a patient had both infectious and respiratory diseases, this patient would not be double calculated (e.g., staphylococcus aureus pneumonia).The clinical characteristics were detailed in Table 1. Respiratory diseases were the main reason for admission to the PICU, and 56% of the patients (702 cases) received mechanical ventilation. The duration of hospitalization in the PICU was 8 (2.1, 16.5) days. The survival group included 1,141 cases (91.1%) while the non-survival group contained 112 cases (8.9%). The survival and non-survival groups showed no significant difference in sex, age, admission diagnosis and length of PICU stay (*P* > 0.05).

**Table 1:** Comparison of clinical data between the survival and non-survival group in PICU.

**Patient characteristics**	**Survival group**	**Non-survival group**	***P***
	**(*n* = 1,141)**	**(*n* = 112)**	
**Sex**			
Male, *n* (%)	752 (65.9)	61 (54.5)	0.406
Female, *n* (%)	389 (34.1)	51 (45.5)	
**Age, years**	3.17 (0.72, 8.98)	3.28 (0.81, 9.02)	0.546
**Age group**			
1 month to <1 year (infants), *n* (%)	218 (19.1%)	26 (23.3%)	0.342
≥1 year and ≤12 years (children), *n* (%)	669 (58.6%)	52 (46.8%)	0.637
≥12 years and ≤14 years (adolescents), *n* (%)	254 (22.3%)	34 (29.9%)	0.445
**Admission diagnostic groups**, ***n*** **(%)**			
Infectious	142 (12.4%)	27 (24.1%)	0.118
Respiratory	287 (25.2%)	40 (35.7%)	0.521
Cancer	193 (16.9%)	12 (10.7%)	0.190
Digestive	113 (9.9%)	9 (8.0%)	0.753
Urological	107 (9.4%)	3 (2.7%)	0.402
Neurological	92 (8.1%)	12 (10.7%)	0.874
Trauma	120 (10.5%)	8 (7.1%)	0.303
Miscellaneous	87 (7.6%)	1 (0.9%)	0.778
**Length of PICU stay, days**	10.5 (3.1, 22.7)	9.1 (2.3, 18.9)	0.073
**Mechanical ventilation**, ***n*** **(%)**	589 (51.6%)	101 (90.7%)	<0.001

Total PRISM III, P-MODS, and PELOD-2 scores in the non-survival group were significantly higher than those in the survival group [PRISM III: 18 (12, 23) vs. 11 (0, 16); PELOD-2: 8 (4, 10) vs. 4 (0, 6); P-MODS: 5 (4, 9) vs. 3 (0, 4), all *P* < 0.001]. The observed mortality in the sample was 8.9% (112/1,253), whereas the mortality rates predicted by PRISM III, PELOD-2, and P-MODS scores were 9.1% (114 deaths), 8.1% (102 deaths), and 7.9% (99 deaths), respectively, in Table 2.

**Table 2:** PRISM III, PELOD-2, and P-MODS scores among critically ill children admitted to PICU.

**Score**	**Non-survival group**	**Survival group**	**χ^2^/*Z***	***P***
PRISM III	18 (12, 23)	11 (0, 16)	60.112	<0.001
PELOD-2	8 (4, 10)	4 (0, 6)	90.313	<0.001
P-MODS	5 (4, 9)	3 (0, 4)	61.978	<0.001

*PRISM III, Pediatric Risk of Mortality III; PELOD−2, Pediatric Logistic Organ Dysfunction 2; P-MODS, Pediatric Multiple Organ Dysfunction Score*.

The discrimination or the ability of the prognostic scoring systems to differentiate between survivors and non-survivors was presented in Table 3. ROC curve analysis showed that the AUCs of PRISM III, PELOD-2, and P-MODS for predicting death of critically ill children were 0.858, 0.721, and 0.596, respectively. Thus, PRISM III showed the best discrimination, followed by PELOD-2 and P-MODS.

**Table 3:** Performance of PRISMIII, PELOD-2, and P-MODS in predicting mortality in critically ill pediatric patients.

**Score**	**AUC**	**Best cutoff**	**Sensitivity (%)**	**Specificity (%)**	**95% CI**	***P***
PRISMIII	0.858	14	73.4	70.7	0.745–0.816	<0.001
PELOD-2	0.721	7	69.1	74.8	0.698–0.766	<0.001
P-MODS	0.596	4	60.3	80.4	0.522–0.613	<0.001

*AUC, Area under curve; CI, confidence interval; PRISM III, Pediatric Risk of Mortality III; PELOD−2, Pediatric Logistic Organ Dysfunction 2; P-MODS, Pediatric Multiple Organ Dysfunction Score*.

In the Hosmer–Lemeshow goodness-of-fit test, PRISM III and PELOD-2 showed the better calibration between the predicted mortality and the observed mortality (PRISM III: χ^2^ = 5.667, *P* = 0.368; PELOD-2: χ^2^ = 9.582, *P* = 0.276; P-MODS: χ^2^ = 12.449, *P* = 0.015), as shown in Table 4.

**Table 4:** The Hosmer-Lemeshow goodness-of-fit test verifying the predicted and the observed outcome of critically ill children by PRISM III and PELOD-2 scores.

**Predicted probability**	**Number of patients**	**Non-survival**		**Survival**	
		**Observed value**	**Predicted value**	**Observed value**	**Predicted value**
**PRISM III**					
0.000–0.552	94	22	21.845	12	12.266
0.553–0.617	91	19	18.048	18	15.427
0.618–0.648	82	17	16.732	10	8.524
0.649–0.706	112	14	12.115	9	7.150
0.707–0.733	121	12	9.336	11	10.378
0.724–0.781	111	16	11.869	8	6.416
0.782–0.802	176	10	6.304	12	10.104
0.803–0.822	165	18	10.447	14	9.007
0.823–0.850	171	9	4.616	9	5.547
0.851–0.999	130	20	11.740	13	6.192
**PELOD-2**					
0.000–0.401	106	18	18.356	15	13.502
0.402–0.592	126	12	12.749	8	6.841
0.593–0.674	124	11	7.641	9	7.002
0.675–0.760	97	10	8.968	6	3.205
0.761–0.819	97	14	7.446	10	8.482
0.820–0.822	100	12	9.337	6	4.001
0.823–0.873	171	9	4.032	14	9.683
0.874–0.907	188	10	5.845	8	3.046
0.908–0.944	138	9	5.403	11	8.997
0.945–0.999	106	12	4.791	10	5.732

*PRISM III, Pediatric Risk of Mortality III; PELOD−2, Pediatric Logistic Organ Dysfunction 2*.

## Discussion

Mortality prediction models are important and valuable tools for assessing the quality of care provided to critically ill patients. Discrimination and calibration of prognostic scores is the most commonly used approach to assess their performance in predicting mortality in critically ill patients, including children. Discrimination is important for distinguishing the outcome, i.e., survival or moribund status, among hospitalized critically ill patients. On the other hand, calibration is more crucial since it allows comparison between predicted and observed outcomes at varying degrees of severity. Although a few studies have used PRISM III and PELOD-2 scores to assess the severity of disease in China (14, 15), there is still no mature and uniformly recognized model for predicting mortality in critically ill pediatric patients. Moreover, the performances of the existing models in Chinese patients were unclear.

In this study, calibration and discrimination of the PRISM III, PELOD-2, and P-MODS scores were assessed and compared in critically ill pediatric patients admitted to PICUs of the First Affiliated Hospital, Sun Yat-Sen University and Shen-Zhen Children's Hospital. A discriminatory ability of 0.80 (AUC) or more was identified as a good tool for discrimination. The closer the ROC curve area was to 1.0, the better the prediction model (16). Both PRISM III (0.858) and PELOD-2 (0.721) met these benchmarks, indicating that PRISM III and PELOD-2 can both discriminate between survival and non-survival. A *P* value more than 0.05 was considered to indicate good calibration. In the Hosmer–Lemeshow goodness-of-fit test, PRISM III and PELOD-2 showed the better calibration between the predicted and observed mortality (PRISM III: χ^2^ = 5.667, *P* = 0.368; PELOD-2: χ^2^ = 9.582, *P* = 0.276; P-MODS: χ^2^ = 12.449, *P* = 0.015). In a cohort study in the United States that enrolled 221 critically ill children in the PICU, PRISM III scores from 0 to 10 corresponded to a mortality rate of 10.2%, while PRISM III scores from 21 to 30 corresponded to a mortality rate as high as 73.8%. Thus, higher PRISM III scores corresponded to higher mortality, indicating that PRISM III assessments can adequately predict the risk of non-survival in critically ill pediatric patients (17). Other studies have also confirmed the good performance of PRISM III (18–22), which was consistent with our research. Therefore, at present, the PRISM III score is considered to be one of the most commonly used tools for predicting mortality in critically ill pediatric patients.

Unlike PRISM III, PELOD-2 is a relatively new tool that has been assessed. PELOD-2 was evaluated in a multicenter cohort study enrolling 3,671 critically ill children (nine PICUs from France and Belgium) (23). In Leteurtre et al.'s study, the PELOD-2 score showed good discrimination (AUC = 0.934, 95% CI, 0.93–0.96). In addition, the Hosmer–Lemeshow goodness-of-fit test also indicated a good calibration (χ^2^ = 9.31, *P* = 0.317). Another study showed that the PELOD-2 score of the non-survival group was significantly higher than that of the survival group. PELOD-2 was confirmed to accurately reflect the severity of organ dysfunction (9). The probability of organ function failure in patients with a PELOD-2 score of 12 or more was higher than that in those with PELOD-2 scores <12 (24, 25). Our results for PELOD-2 performance were consistent with those reported previously. In our study, the AUC of the PELOD-2 score seems lower than the AUC reported by the French and Belgium study (0.721 vs. 0.934). This may be due to differences in different regions and races. Meanwhile, the value of 0.721 is also acceptable.

The overall mortality in our study was 8.9%, which was significantly lower than the high mortality in the early days of PICU (12.8%) (26), but it was still higher than the rates in European PICUs (about 5.2%) (27) and PICUs in the US (~2.5%) (28). The higher mortality rates in Chinese PICUs in the present study can be attributed to various reasons. First, the characteristics of PICU patients in this study were certainly different from those in western countries, with severe pneumonia, the leading cause of death, accounting for approximately one-third of the deaths. Second, broad-spectrum antibiotics were frequently used to rapidly control bacterial infection, which may have led to the generation of multidrug resistant bacteria or new bacterial infections. Third, in comparison with developed countries, China has a relatively less developed medical infrastructure, and much effort will be required to improve the infrastructure in our country.

Due to the complexity of the disease, assessment of the illness condition and prediction of the risk of death is a complicated project. Therefore, there is no consensus on the gold standard score for such assessments. In this study, we found that PRISM III and PELOD-2 offered good discrimination in two Chinese PICUs. PRISM III and PELOD-2 showed the better calibration between predicted and observed mortality, while P-MODS showed poor calibration. The results of this study are helpful to effectively and accurately evaluate the severity of critically ill children in two Chinese PICUs. Furthermore, through enrolling a huge number of Chinese PICU patients in the further study, in-depth effective predictive factors could be excavated and a more credible and accurate predictive model suitable for PICU in China could be established.

### Limitations

This study had several limitations. First, given the nature of the study, the quality of recorded data could threaten the validity of the findings. However, the variables prospectively collected for PRISM III included some essential clinical information that was also used for PELOD-2 estimation. Second, only two PICU centers were enrolled in this study, and the number of patients was not large enough to be sufficiently representative of the Chinese PICU population. Thus, to obtain more reliable and exact results, future studies should extend this study to more Chinese PICUs. Third, PELOD-2 and P-MODS are designed as a surrogate marker for mortality and used to assess organ failure on all PICU days. However, all indicators in this study were collected within 24 h of admission and there was no dynamic evaluation of the predictive value of PELOD-2 and P-MODS.

### Conclusion

In this study, we found that PRISM III and PELOD-2 offered good discrimination in two Chinese PICUs. PRISM III and PELOD-2 showed the better calibration between predicted and observed mortality, while P-MODS showed poor calibration. The results of this study contribute to evaluating the condition of critically ill pediatric patients rapidly and exactly. Moreover, more in-depth research needs to be carried out in the future to explore more reliable and accurate predictive model propitious to PICUs in China.

## Data Availability Statement

The raw data supporting the conclusions of this article will be made available by the authors, without undue reservation.

## Ethics Statement

The procedures involving human participants were reviewed and approved by Institutional Review Board of the First Affiliated Hospital and the Seventh Affiliated Hospital, Sun Yat-Sen University, and Shen-Zhen Children's Hospital. Written informed consent to participate in this study was provided by the participants' legal guardian/next of kin.

## Author Contributions

WT and XL conceptualized and designed the study and reviewed and revised the manuscript. LZ coordinated and supervised data collection, drafted the initial manuscript, and reviewed and revised the manuscript. YW carried out the analyses, interpreted the results, drafted the initial manuscript, and reviewed and revised the manuscript. HH, CL, YC, and LX contributed to data collection and reviewed and revised the manuscript. All authors contributed to manuscript revision and read and approved the submitted version.

## Conflict of Interest

The authors declare that the research was conducted in the absence of any commercial or financial relationships that could be construed as a potential conflict of interest.
